# Genetic diversity analysis of phenotypic and agronomic traits in oat germplasm resources

**DOI:** 10.3389/fpls.2025.1670684

**Published:** 2025-09-02

**Authors:** Yan Zhao, Jiaqi Fang, René Gislum, Baowen Zhao, Zhiming Zhong, Yingxia Lei, Donghai Yan, Ruzhi He, Youjun Chen, Qingping Zhou, Hui Wang

**Affiliations:** ^1^ Sichuan Zoige Alpine Wetland Ecosystem National Observation and Research Station, Southwest Minzu University, Chengdu, China; ^2^ Department of Agroecology, Aarhus University, Slagelse, Denmark; ^3^ Institute of Geographic Sciences and Natural Resources Research, Chinese Academy of Sciences, Beijing, China; ^4^ Grassland Technology Research and Extension Center of Sichuan Province, Chengdu, China; ^5^ Gansu Puruituo (PRT) Eco-agricultural Science and Technology Co., Ltd., Zhangye, China

**Keywords:** *Avena sativa*, resource evaluation, principal component analysis, structural equation modeling, quantitative trait

## Abstract

The evaluation of genetic diversity in germplasm resources is fundamental to crop breeding. A total of 183 oat germplasm resources were evaluated through field trials at Xinjin District and Shandan County, located in southern and northern China, respectively. Phenotypic and agronomic traits were assessed, including six qualitative and sixteen quantitative characteristics. Results revealed significant variation in panicle attitude and grain color, based on the statistical analysis using SPSS. Among the sixteen quantitative traits, coefficient of variation ranged from 4.92% to 48.02% with the second internode thickness exhibiting the highest genetic diversity index. Correlation analysis of sixteen quantitative traits was performed using R Studio, and the results indicated significant positive relationships between grain weight and several ear characteristics, including spikelet number, ear length, layer numbers, and grain numbers per ear. Principal component analysis categorized the sixteen quantitative phenotypic traits into five independent factors. The structural equation modeling using SPSS-AMOS indicated that ear characteristics showed strong direct contributions to grain weight, establishing it as a key indicator for future breeding efforts. The multiple correspondence analysis by R Studio suggested that a total of nineteen oat germplasm resources showed the grain and biomass production potential across both experimental regions.

## 1 Introduction

Oats (*Avena sativa*), an annual crop in the Gramineae family, comprise two main types: hulled oats and hulless oats (Ren and Hu, 2013; Peng et al., 2022). Hulless oat grains are valued as a nutrient-dense food source due to their high nutritional value, including elevated levels of protein, fat, β-glucan, and essential minerals (Rafique et al., 2022). Their consumption has been associated with health benefits, such as reduced cholesterol levels and a lower risk of coronary heart disease and cancer (FDA, 2023). Meanwhile, immature hulled oat plants serve as high-quality forage, prized for their tenderness, high nutrient content, and palatability, making them well-suited for green hay and silage production (Zhao et al., 2018; Canales et al., 2021). Oats are widely cultivated owing to their strong stress tolerance and broad adaptability. Additionally, they play a crucial role in restoring degraded natural grasslands and improving ecological sustainability (Yao et al., 2024).

Oats are cultivated in more than 40 countries worldwide, primarily within the Northern Hemisphere oat belt—a region encompassing Asia, Europe, and North America above 40° north latitude. Significant oat production also occurs in Southern Hemisphere countries such as Australia, New Zealand, and Brazil. From 2009 to 2013, the global average oat cultivation area reached approximately 9.6 million hectares (Gorash et al., 2017). According to the Food and Agriculture Organization of the United Nations (FAO), nearly 130,000 oat germplasm resources have been collected and preserved globally, comprising 24% wild materials, 14% landraces, 13% breeding lines, 12% improved varieties, and 37% other types (Ren and Hu, 2013; Menon et al., 2016). Countries such as Canada, the United States, Russia, Germany, and Kenya maintain particularly extensive collections of oat germplasm. Leveraging crop diversity is critical for enhancing agricultural productivity and ensuring global food security (Teklu et al., 2006). The loss of crop genetic diversity could lead to widespread crop failures across major production regions (Khoury et al., 2022). Breeding efficiency can be significantly improved by utilizing parental lines with diverse genetic backgrounds (Livanios et al., 2018). Furthermore, comprehensive research on crop genetic diversity not only supports the conservation of germplasm resources but also facilitates the introduction of elite traits into modern cultivars, thereby enhancing cultivation potential (Mengistu et al., 2015).

Cultivated oat populations exhibit greater genetic diversity than wheat and barley (O’Donoughue et al., 1994), enabling broader environmental adaptation and versatile end-use applications. Investigating the genetic diversity of oat germplasm resources, along with phenotypic variation and trait heritability, facilitates the screening of elite germplasm, identification of functional genes, and development of efficient breeding strategies (Cieplak et al., 2021). Such research also supports the cultivation of improved oat varieties that meet market and consumer demands (Tinker et al., 2022; Yan et al., 2020; Herrmann et al., 2014). For instance, Wang et al. (2024) analyzed 590 oat accessions and found that the third internode thickness and stem node pubescence exhibited the highest genetic diversity, whereas plant size and flag leaf area showed the lowest variability. In China’s Hexi Corridor, key agronomic traits influencing oat growth, development, and yield included plant height, ear length, and hay yield across 27 cultivars (Wang et al., 2023). Similarly, Mathias-Ramwell et al. (2023) observed significant variation (H’≥ 0.7) in plant height and seed yield among 132 oat germplasm accessions, highlighting their high diversity. Additionally, prior studies confirmed that yield-related traits—such as spikelet numbers, seed set rate, floret numbers, and ear characteristics—are major determinants of seed yield (Zhan et al., 2024; Ju et al., 2022; Doehlert et al., 2002).

Long-term selective breeding has led to the development of diverse oat ecotypes, with significant variations observed in agronomic traits, seed morphology, and yield performance under different environmental conditions (Zhou et al., 2021). Cultivar introduction trials across various regions have further revealed substantial differences in agronomic characteristics and quality traits among oat varieties grown in distinct ecological environments, highlighting the importance of comprehensive multi-environment evaluation of oat germplasm (Alexander et al., 2025; Mut et al., 2022). This study evaluated 183 oat accessions through a comprehensive analysis of phenotypic and agronomic traits, and characterized the genetic diversity of these resources. The findings provide a scientific foundation for oat germplasm enhancement and innovative breeding strategies.

## 2 Materials and methods

### 2.1 Experimental materials

**Figure 1:**
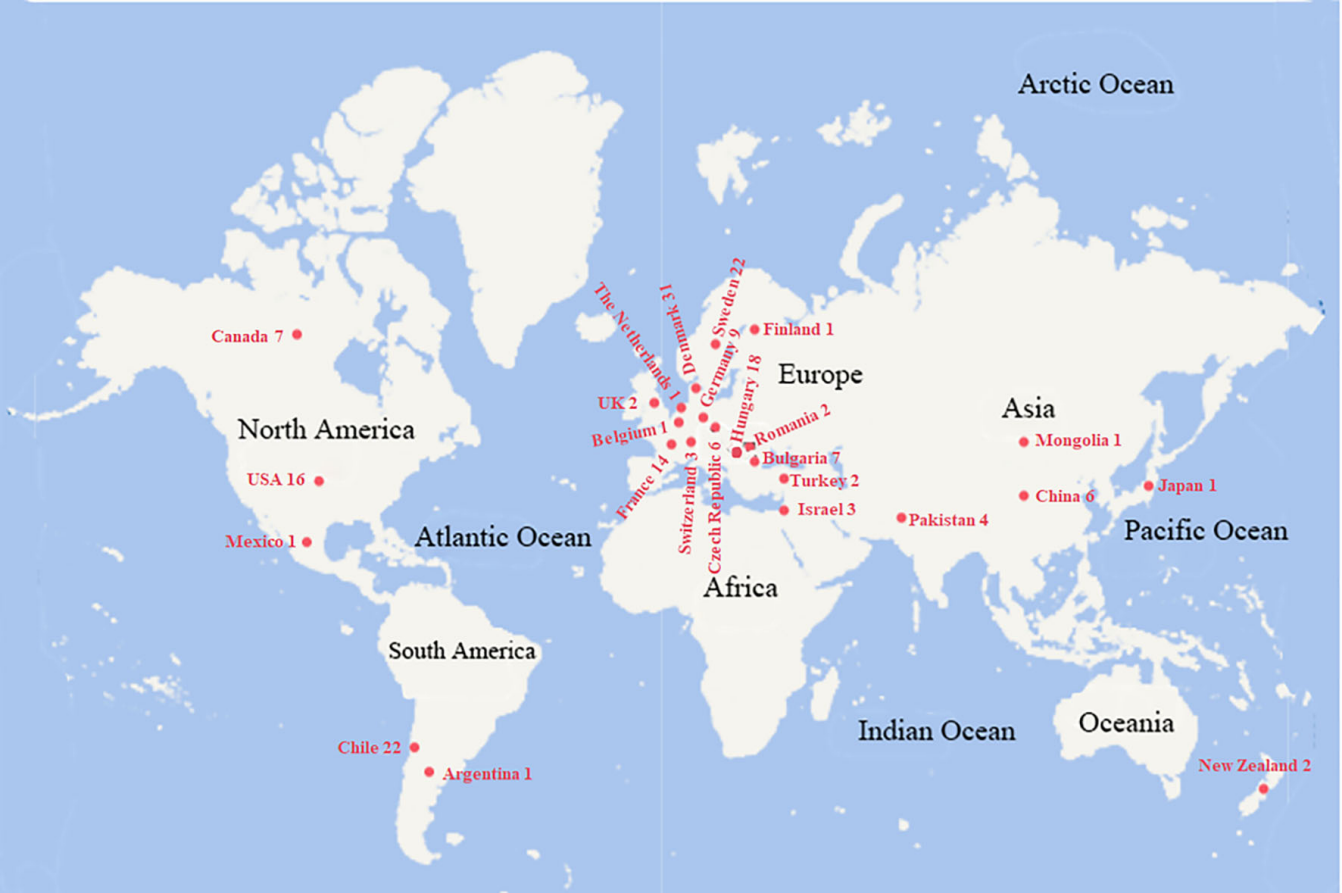
The origin country of the tested oat germplasm resources. The number after the country name is the resource amount from the corresponding origin.

This study evaluated 183 oat germplasm accessions sourced from three institutions: the National Animal Husbandry Service of China, the Institute of Crop Sciences at the Chinese Academy of Agricultural Sciences, and Southwest Minzu University. These accessions originated from 26 countries worldwide, representing a diverse geographical distribution (**Figure 1**).

### 2.2 Experimental sites

**Figure 2:**
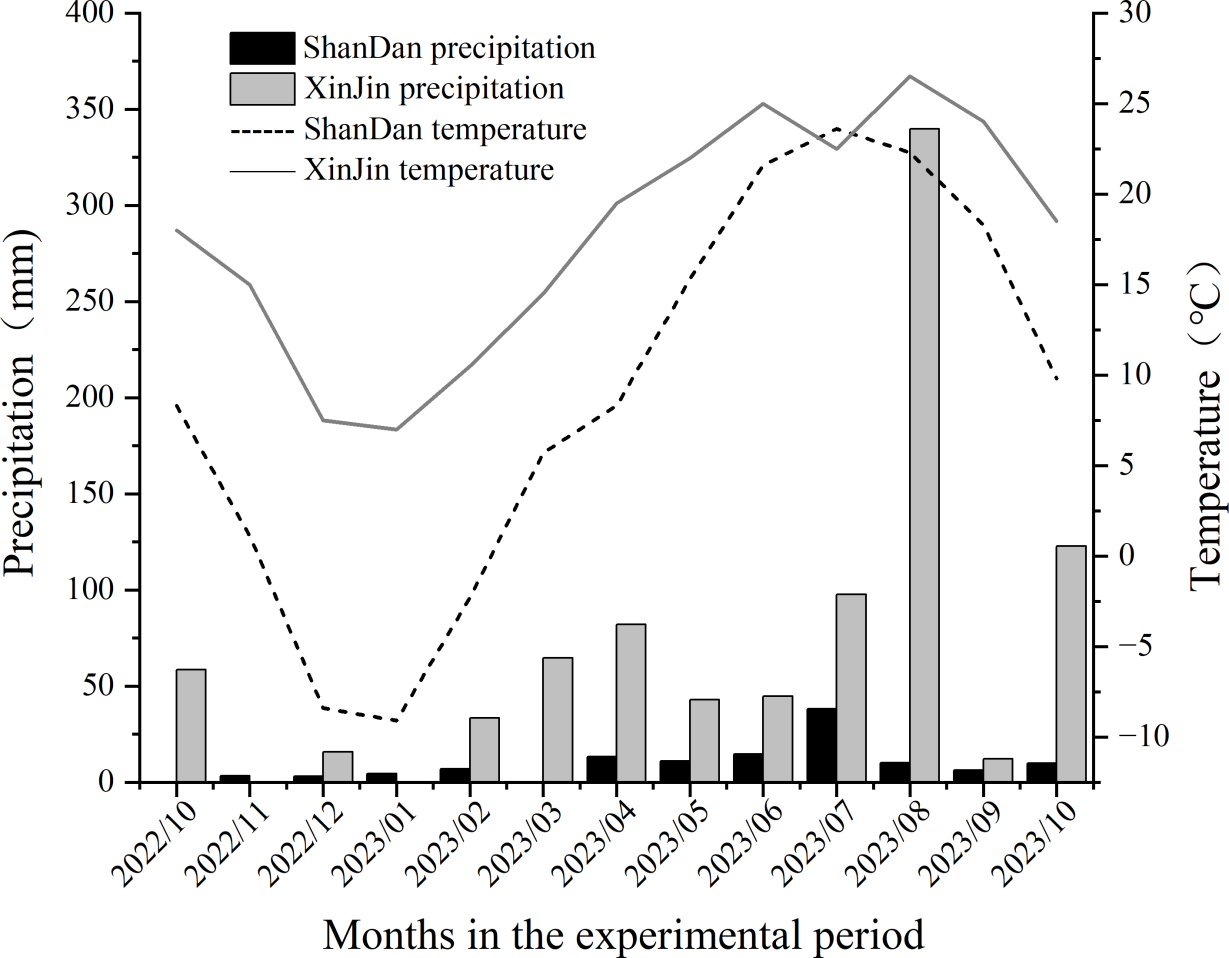
Monthly mean temperature and total precipitation distribution in Shandan and Xinjin during 2022-2023.

Field experiments were conducted from October 2022 to May 2023 at two locations: (1) Yulong Village, Baodun Town, Xinjin District, Chengdu City, Sichuan Province (30°28’N, 108°45’E), and (2) Xipo Village, Huocheng Town, Shandan County, Zhangye City, Gansu Province (38°40’N, 101°05’E). Xinjin District features a subtropical humid monsoon climate, characterized with an average elevation of 478 m, a mean annual temperature of 16.4°C, annual precipitation of 987 mm, and a frost-free period averaging 297 days. In contrast, Shandan County exhibits a continental arid climate, with an average elevation of 2,456 m, a mean annual temperature of 7.0°C, annual precipitation of 230 mm, and a shorter frost-free period of 123 days. During the 2022–2023 study period, significant climatic differences were observed between the two sites, with Xinjin demonstrating consistently higher temperatures and greater precipitation compared to Shandan (**Figure 2**).

### 2.3 Experimental design

The oat germplasm seeds were sown on October 26, 2022, in Xinjin District and on April 26, 2023, in Shandan County. The seeds were drill-planted with 50 cm row spacing at a sowing rate of 5 g/m, with each accession planted in a 3 m row. Protective borders were established around the experimental plots, and field management followed standard local cultivation practices.

### 2.4 Trait investigation

For morphological trait evaluation, five to ten oat plants were randomly selected following the standardized protocols from (Liu et al., 2013; UPOV, 2018). Qualitative traits, including grain form, grain color, hull or hulless, panicle direction, panicle attitude, and flag leaf attitude, were observed, and the diversity index and frequency distribution of each trait were calculated. Quantitative traits included 16 indicators: growth duration, branch node numbers, blade numbers, the second internode thick, the second internode length, internode length below spike, plant height, flag leaf length, flag leaf width, tiller numbers, fertile tiller numbers, ear length, layer numbers, spikelet numbers, grain number per ear and grain weight.

Qualitative traits were given values according to the released Guidelines. Quantitative traits were classified following Nan et al. (2017). Genetic diversity was assessed using the Shannon-Wiener’s diversity index, which is determined using the formula H’ =-Σ*P_i_* (*lnP_i_*), where *P_i_* denotes the frequency of a particular feature at level *i* in the sample.

Each quantitative trait of the tested germplasm’s overall mean (X) and standard deviation (σ) was calculated. The genetic diversity index was then calculated using the frequency of each grade, which was categorized as follows: 1 grade ≤ *X*-2*σ*, 10 grade > *X*+2*σ*, *i*-1 < *i* <= *i*+1 (*i* indicates the number of grades, i = 2-9), with a 0.5σ interval between each grade.

The following formula was used to classify the maturity of 183 oat germplasm resources into extra-early maturity, early maturity, medium maturity, late maturity, and extra-late maturity. *D* = *μ* ± *kσ*, where *k* = 1 or 2, μ is the average value of the growth period of each oat resource, and σ is the standard deviation.

### 2.5 Data statistics

Data preprocessing was performed using Microsoft Excel 2010. Basic statistical analyses, including calculation of means, standard deviations, range values (minimum and maximum), and coefficients of variation, were conducted using SPSS 25.0. Correlation matrices were generated and visualized using the *corrplot* package in R (version 4.1.0), and the relevance and proximity of the correlation between the indicators were explained. Cluster analysis was performed employing Ward’s minimum variance method via the *cluster* package to optimize both within-group homogeneity and between-group heterogeneity. Principal component analysis aims to scientifically combine several indicators, understand the overall morphological traits of crops, and efficiently streamline the analysis processes. The principal component analysis of agronomic traits was carried out using SPSS 25.0 software. Structural equation modeling was implemented using IBM AMOS 29.0. Using the *dplyr* package in R Studio, the raw data was binned into three categories (high, medium, low) based on quantiles. The multiple correspondence analysis was performed with the *FactoMineR* package to calculate coordinates and contribution rates. Visualize the results using the *factoextra* package, and conduct clustering based on the multiple correspondence analysis results.

## 3 Results

### 3.1 Genetic diversity analysis

Most of the 183 oat germplasm resources were characterized as yellow grain (frequency=0.72), spindle-shaped grains (frequency=0.85), hulled oats (frequency=0.98), erect flag leaves (frequency=0.90), peripheral panicles (frequency=0.94), and erect ear (frequency=0.46) (**Table 1**). Shannon-Wiener’s diversity index of grain color and panicle attitude exceeded 1, which implied that these 183 oat germplasm resources had rich variability and a large genetic foundation.

**Table 1:** Genetic diversity of qualitative traits in oat germplasm resources.

Qualitative traits	H’	Frequency								
		1	2	3	4	5	6	7	8	9
Grain color	1.134	0.02	0.72	0.05	0.01	0.03	0.03	0.08	0.02	0.04
Grain shape	0.495	0.13	0.85	0.02						
Hull or hulless	0.112	0.98	0.02							
Flag leaf attitude	0.267	0.90	0.04	0.06						
Panicle direction	0.389	0.04	0.02	0.94						
Panicle attitude	1.2	0.23	0.46	0.25	0.06					

Grain color: 1-light yellow, 2-yellow, 3-white, 4-red-yellow, 5-red, 6-brown-yellow, 7-brown, 8-black-yellow, 9-black; Grain shape: 1-ellipsoid, 2-fusiform, 3-lanceolate; Hull/hulless: 1-hull, 2-hulless; Flag leaf attitude:1-upward, 2-horizontal, 3-drooping; Panicle direction:1-unilateral, 2-central, 3-peripheral; Panicle attitude:1-erect, 2-semi-erect, 3-horizontal, 4-downward.

In Shandan County, six traits exhibited significant variation: spikelet numbers (48.0%), grain weight (46.6%), grains per panicle (43.6%), fertile tiller count (41.80%), total tiller numbers (41.3%), and second internode length (39.3%) (**Table 2**). These high coefficients of variation (CV) indicate substantial phenotypic diversity for these traits under Shandan’s growing conditions, offering valuable selection potential for varietal improvement. Similarly, in Xinjin District, five traits showed particularly high variability: grain weight and total tiller numbers (both 40.9%), fertile tiller count (38.0%), grains per panicle (37.3%), and spikelet numbers (36.8%) (**Table 2**). In contrast, growth duration displayed the lowest CV at both locations (Shandan: 4.92%; Xinjin: 5.64%), reflecting its relative stability across genotypes. However, absolute growth duration differed substantially between sites, ranging from 82–103 days in Shandan County compared to 105–163 days in Xinjin District, reflecting differences in sowing dates and climatic conditions. Based on growth duration, the 183 accessions were classified into four or five distinct maturity groups (**Table 3**). Notably, the second internode diameter showed the highest Shannon-Wiener diversity index (H’) values in both environments (Shandan: 2.08; Xinjin: 2.07) (**Table 2**). Overall, the materials exhibited significant genetic variation across quantitative traits, demonstrating both a broad genetic base and rich diversity for breeding applications.

**Table 2:** Genetic variation of quantitative traits of oat germplasm.

Experimental sites	Quantitative traits	Min.	Max.	Mean	SD	CV (%)	*H′*
Shandan County	Growth duration (d)	82.0	103	90.3	4.44	4.92	2.00
	Branch node numbers (no.)	3.00	7	4.83	0.75	15.5	1.13
	Blade numbers (no.)	3.00	7	4.50	0.74	16.4	1.09
	The second internode thickness (mm)	1.40	9.70	5.24	1.12	21.4	2.08
	The second internode length (cm)	2.70	32.50	11.2	4.39	39.3	2.00
	Internode length below ear (cm)	15.6	98.1	40.6	8.01	19.8	2.04
	Plant height (cm)	38.0	143.8	98.0	17.0	17.4	2.07
	Flag leaf length (cm)	6.50	37.0	17.3	4.69	27.1	2.05
	Flag leaf width (cm)	0.30	3.0	1.56	0.40	25.6	2.07
	Tiller numbers (no.)	2.00	26	8.45	3.49	41.3	1.94
	Fertile tiller numbers (no.)	1.00	20	6.65	2.78	41.8	1.93
	Ear length (cm)	9.80	43.3	21.3	5.53	26.0	2.05
	Layer numbers (no.)	3.00	8	5.41	0.95	17.6	1.36
	Spikelet numbers (no.)	5.00	148	48.5	23.3	48.0	2.04
	Grain numbers per ear (no.)	10.0	302	99.4	43.3	43.6	2.05
	Grain weight (g)	0.13	10.54	3.48	1.62	46.6	2.05
Xinjin District	Growth duration (d)	105.	163	136	7.71	5.64	1.63
	Branch node s (no.)	5.00	10.0	7.20	0.91	12.6	1.32
	Blade numbers (no.)	4.00	10.0	6.74	0.98	14.6	1.39
	The second internode thickness (mm)	1.25	10.8	6.67	1.34	20.1	2.07
	The second internode length (cm)	2.40	20.6	10.1	3.41	33.9	2.06
	Internode length below ear (cm)	1.10	89.0	33.9	9.12	26.9	2.04
	Plant height (cm)	88.0	224.	141	22.4	15.9	2.06
	Flag leaf length (cm)	2.00	48.0	21.5	7.19	33.5	2.02
	Flag leaf width (cm)	1.20	5.20	2.76	0.72	26.0	2.04
	Tiller numbers (no.)	3.00	46.0	11.9	4.88	40.9	1.98
	Fertile tiller numbers (no.)	2.00	20.0	7.56	2.87	38.0	1.92
	Ear length (cm)	10.8	83.0	35.1	9.68	27. 6	1.96
	Layer numbers (no.)	4.00	12.00	8.47	1.16	13.8	1.53
	Spikelet numbers (no.)	28.0	291.	98.4	36.2	36.8	2.01
	Grain numbers per ear (no.)	45.0	544.	177	66.2	37.3	1.99
	Grain weight (g)	0.45	12.19	4.02	1.65	40.9	2.00

**Table 3:** Classification of maturity of test germplasm.

Experiment sites	Maturity	Classification standard	Amount (no.)	Mean (d)	SD
Shandan	Early maturity	81<D ≤ 85	29	83.9	1.24
	Medium maturity	85<D ≤ 94	116	89.7	2.10
	Late maturity	94<D ≤ 99	37	96.9	1.33
	Extra-late maturity	D>99	1	103	0.00
Xinjin	Extra-early Maturity	D ≤ 120	3	105.00	0.00
	Early maturity	120<D ≤ 128	33	126.97	1.65
	Medium maturity	128<D ≤ 144	126	137.38	2.29
	Late maturity	144<D ≤ 152	9	147.11	0.33
	Extra-late maturity	D>152	12	154.25	3.11

### 3.2 Correlation analysis of quantitative traits

**Figure 3:**
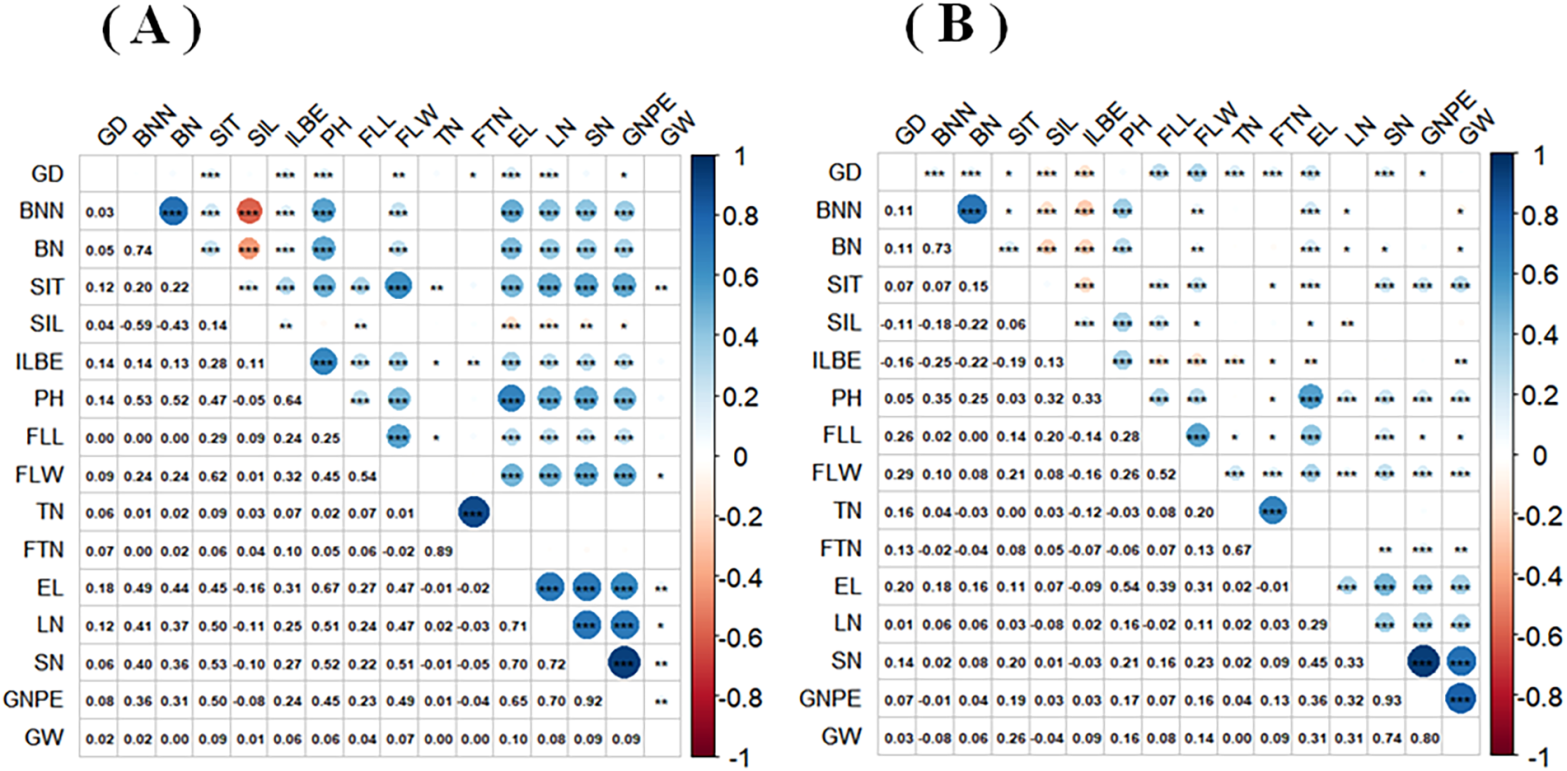
Correlation of 16 quantitative traits of oat germplasm resources grown in Shandan County **(A)** and Xinjin District **(B)**. *P<0.05; **P<0.01; ***P<0.001. GD, Growth duration; BNM, Branch node numbers; BN, Blade numbers; SIT, The second internode thickness; SIL, The second internode length; ILBE, Internode length below ear; PH, Plant height; FLL, Flag leaf length; FLW, Flag leaf width; TN, Tiller numbers; FTN, Fertile tiller numbers; EL, Ear length; LN, Layer numbers; SN, Spikelet numbers; GNPE, Grain numbers per ear; GW, Grain weight.

Correlation analyses revealed varying degrees of association among oat traits in both cultivation regions, with most trait correlations reaching highly significant levels (P<0.001, **Figure 3**). Notably, ear length showed strong positive correlations (P<0.001) with other ear characteristics. The strongest pairwise correlations were observed between spikelet numbers and grain numbers, with correlation coefficients of 0.93 and 0.92 in the two regions, respectively. As most of the 16 quantitative traits exhibited significant intercorrelations, we performed principal component analysis to reduce data dimensionality and mitigate potential multicollinearity effects among variables.

### 3.3 Cluster analysis of oat agronomic traits

**Figure 4:**
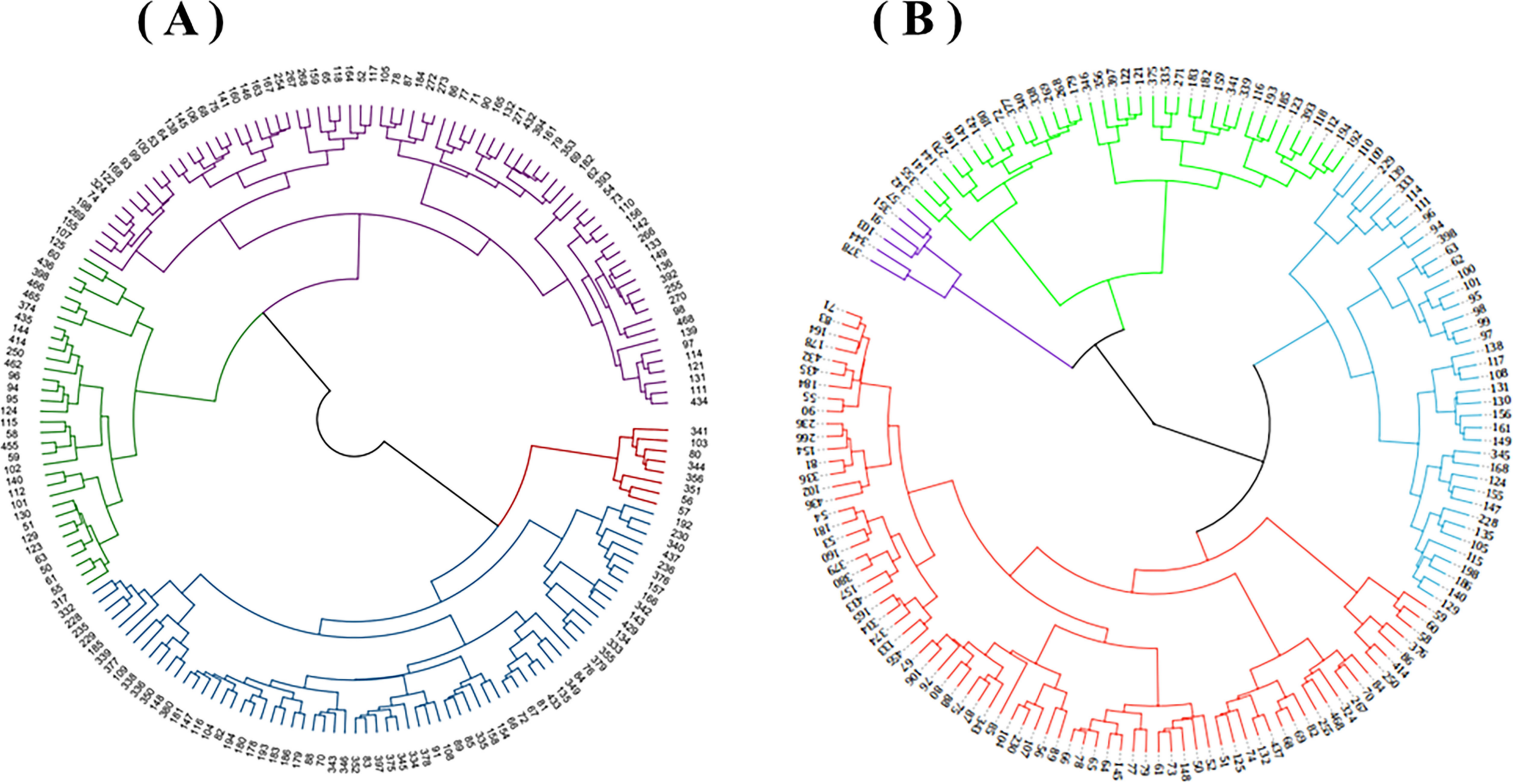
Cluster analysis of 183 oat germplasm grown in Shandan County **(A)** and Xinjin District **(B)**.

Based on the clustering analysis, 183 oat germplasm resources were clustered into four groups (**Figure 4**). The four germplasm groups were then statistically analyzed and compared to gain a thorough understanding of the genetic relationships of the tested germplasm resources and to elucidate the type differences among the germplasm.

The 8 resources, accounting for 4.4% of the total tested materials, were in Shandan County’s Germplasm Group I (**Table 4**). These resources displayed medium-late maturity, high height, high yield, and long branch internodes. Germplasm Group II contained 67 resources, accounting for 36.6%, had a long growth period, high stalks, rich blades, and long and thick second internodes. The 33 resources in Germplasm Group III, accounting for 18%, demonstrated that the plants were short, strong-tillering, and poor-yielding. Germplasm Group IV contained 75 resources, accounting for 41%, and were displayed as early-maturity, medium-height, and moderate-production.

**Table 4:** Results of comparison of mean values of quantitative traits of germplasm resources of different taxa.

Experimental sites	Quantitative traits	Taxa I	Taxa II	Taxa III	Taxa IV
Shandan County	Growth duration (d)	90.8 ± 7.23^a^	91.6 ± 4.48^a^	90.1 ± 4.53^a^	89.1 ± 3.70^a^
	Branch node numbers (no.)	4.68 ± 0.47^b^	5.20 ± 0.55^a^	4.28 ± 0.43^c^	4.73 ± 0.42^b^
	Blade numbers (no.)	4.50 ± 0.28^b^	4.87 ± 0.49^a^	3.95 ± 0.40^c^	4.38 ± 0.42^b^
	The second internode thickness (mm)	5.87 ± 0.42^a^	5.88 ± 0.65^a^	4.00 ± 0.63^c^	5.13 ± 0.69^b^
	The second internode length (cm)	12.0 ± 3.22^a^	11.0 ± 2.84^a^	11.5 ± 2.49^a^	11.4 ± 3.28^a^
	Internode length below ear (cm)	46.2 ± 5.07^a^	43.9 ± 5.22^a^	35.0 ± 6.23^c^	39.4 ± 5.48^b^
	Plant height (cm)	111 ± 5.53^a^	111 ± 11.0^a^	79.3 ± 10.1^c^	93.1 ± 10.1^b^
	Flag leaf length (cm)	19.5 ± 2.11^a^	18.2 ± 3.07^a^	13.8 ± 2.85^b^	17.8 ± 2.72^a^
	Flag leaf width (cm)	1.86 ± 0.14^a^	1.77 ± 0.24^a^	1.10 ± 0.25^c^	1.54 ± 0.21^b^
	Tiller numbers (no.)	7.18 ± 1.82^a^	8.74 ± 2.55^a^	8.02 ± 3.52^a^	8.00 ± 2.35^a^
	Fertile tiller numbers (no.)	5.85 ± 1.33^b^	6.88 ± 2.04^ab^	7.08 ± 2.26^a^	6.36 ± 1.47^ab^
	Ear length (cm)	26.2 ± 2.48^a^	25.7 ± 3.93^a^	14.9 ± 2.09^c^	196 ± 3.40^b^
	Layer numbers (no.)	5.95 ± 0.46^a^	6.08 ± 0.43^a^	4.20 ± 0.53^c^	5.25 ± 0.58^b^
	Spikelet numbers (no.)	81.1 ± 15.3^a^	64.6 ± 15.6^b^	21.4 ± 10.6^d^	42.3 ± 13.7^c^
	Grain numbers per ear (no.)	135 ± 12.5^a^	129 ± 28.2^a^	504 ± 22.9^c^	90.2 ± 30.2^b^
	Grain weight (g)	4.50 ± 0.95^a^	4.52 ± 1.06^a^	1.70 ± 0.87^c^	3.22 ± 1.35^b^
Xinjin District	Growth duration (d)	134 ± 9.07^b^	138 ± 7.86^b^	137 ± 2.58^b^	150 ± 10.07^a^
	Branch node numbers (no.)	7.10 ± 0.74^b^	7.11 ± 0.62^b^	7.38 ± 0.61^ab^	7.73 ± 1.04^a^
	Blade numbers (no.)	6.69 ± 0.78^b^	6.64 ± 0.68^b^	6.93 ± 0.61^b^	7.72 ± 1.14^a^
	The second internode thickness (mm)	6.37 ± 1.04^b^	6.85 ± 0.89^b^	6.95 ± 0.87^b^	8.14 ± 0.58^b^
	The second internode length (cm)	8.95 ± 2.64^b^	10.4 ± 2.2^ab^	11.1 ± 2.79^a^	8.81 ± 1.84^b^
	Internode length below ear (cm)	36.1 ± 7.27^a^	30.7 ± 5.67^b^	35.4 ± 4.33^a^	23.4 ± 13.4^c^
	Plant height (cm)	138 ± 14.8^bc^	130 ± 16.6^c^	157 ± 21.4^a^	146 ± 16.6^ab^
	Flag leaf length (cm)	19.5 ± 4.19^b^	21.2 ± 5.67^b^	22.8 ± 5.52^b^	35.6 ± 6.59^a^
	Flag leaf width (cm)	2.51 ± 0.49^c^	2.70 ± 0.57^c^	3.09 ± 0.48^b^	4.02 ± 0.47^a^
	Tiller numbers (no.)	9.98 ± 2.25^c^	15.1 ± 4.22^a^	12.6 ± 3.19^b^	8.22 ± 2.9^c^
	Fertile tiller numbers (no.)	6.59 ± 1.14^c^	9.60 ± 1.66^a^	7.77 ± 1.94^b^	6.25 ± 1.66^c^
	Ear length (cm)	32.9 ± 6.16^b^	30.6 ± 5.26^b^	40.7 ± 8.35^a^	44.2 ± 7.51^a^
	Layer numbers (no.)	8.54 ± 0.77^ab^	8.17 ± 0.71^b^	8.81 ± 0.92^a^	8.32 ± 0.71^ab^
	Spikelet numbers (no.)	87.1 ± 22.1^b^	90.2 ± 224^b^	123 ± 26.2^a^	120 ± 34.7^a^
	Grain numbers per ear (no.)	157 ± 36.1^b^	167 ± 37.3^b^	223 ± 50.5^a^	185 ± 78.7^b^
	Grain weight (g)	3.72 ± 1.03^b^	3.65 ± 0.88^b^	5.08 ± 1.45^a^	4.68 ± 2.23^a^

Different letters within the same row mean a significant difference at the 0.05 probability level.

Germplasm Group I contained 76 resources, accounting for 47.8% of the total evaluated materials in Xinjin District. These resources displayed early maturity, short and narrow flag leaves, and low grain yield. Germplasm Group II contained 39 resources, accounting for 24.5%, had medium-late maturity, short plants, and strong tillering capacity. The 38 resources, accounting for 23.9%, were in Germplasm Group III. These resources displayed tall plants, thick stalks, and high grain yield. Germplasm Group IV contained 6 resources, accounting for 3.8%, had medium stalks, late maturity, large spikes, and long and wide flag leaves.

### 3.4 Principal component analysis of agronomic traits in oat

The eigenvalue over one was used as the extraction principle, and five principal components, including ear characteristics, blade numbers, tiller numbers, flag leaf size, and stem length, were extracted as independent factors (**Table 5** and **Table 6**). These five principal components above could be the new cultivar breeding objectives for grain or biomass production. The principal component in ear characteristics, containing spikelet numbers, grain numbers per ear, layer numbers, and ear length, is associated with grain yield. The principal components in blade numbers, containing branch node numbers and blade numbers, and in the flag leaf size, containing flag leaf length and width, represent the amount of leaves, which contribute to the hay quality of oat. In addition, the principal components in tiller numbers, containing tiller and fertile tiller numbers, and in stem length, containing internode length below spike, internode length below spike, and plant height, are essential for biomass production. The cumulative contribution rate of these five principal components in the two planting regions was 76.657% and 66.347%, respectively.

**Table 5:** Principal component analysis of quantitative traits of oat germplasm in Shandan County.

Quantitative traits	Principal components				
	PC 1	PC 2	PC 3	PC 4	PC 5
Growth duration (d)	0.14	-0.09	0.07	-0.34	0.67
Branch node numbers (no.)	0.29	0.87	0.01	0.03	0.13
Blade numbers (no.)	0.27	0.79	0.02	0.04	0.18
The second internode thickness (mm)	0.64	-0.06	0.09	0.37	0.20
The second internode length (cm)	0.02	-0.81	0.03	0.10	0.22
Internode length below spike (cm)	0.10	0.04	0.03	0.41	0.72
Plant height (cm)	0.41	0.37	0.00	0.34	0.64
Flag leaf length (cm)	0.16	-0.07	0.05	0.81	0.02
Flag leaf width (cm)	0.52	0.06	-0.02	0.64	0.12
Tiller numbers (no.)	0.02	0.00	0.97	0.03	0.01
Fertile tiller numbers (no.)	-0.04	0.00	0.97	0.03	0.06
Ear length (cm)	0.72	0.32	-0.03	0.15	0.29
Layer numbers (no.)	0.82	0.20	-0.01	0.10	0.14
Spikelet numbers (no.)	0.92	0.16	-0.03	0.10	0.06
Grain numbers per ear (no.)	0.91	0.12	-0.01	0.08	0.02
Eigenvalue	5.42	2.04	1.84	1.16	1.03
Proportion/%	36.2	13.6	12.3	7.75	6.88
Cumulative proportion/%	36.2	49.8	62.0	69.8	76.7

**Table 6:** Principal component analysis of quantitative traits of oat germplasm in Xinjin District.

Quantitative traits	Principal components				
	PC 1	PC 2	PC 3	PC 4	PC 5
Growth duration (d)	0.05	0.63	0.07	0.13	-0.35
Branch node numbers (no.)	-0.01	0.06	0.92	0.03	-0.03
Blade numbers (no.)	0.05	0.01	0.89	-0.04	-0.10
The second internode thickness (mm)	0.14	0.09	0.09	0.00	0.08
The second internode length (cm)	-0.10	0.09	-0.22	0.08	0.76
Internode length below spike (cm)	0.11	-0.25	-0.20	-0.08	0.52
Plant height (cm)	0.23	0.30	0.43	-0.08	0.70
Flag leaf length (cm)	-0.02	0.80	-0.05	-0.01	0.20
Flag leaf width (cm)	0.12	0.74	0.04	0.14	0.07
Tiller numbers (no.)	-0.01	0.13	0.02	0.90	-0.02
Fertile tiller numbers (no.)	0.08	0.03	-0.03	0.91	0.01
Ear length (cm)	0.48	0.53	0.24	-0.10	0.23
Layer numbers (no.)	0.60	0.02	0.11	0.02	-0.07
Spikelet numbers (no.)	0.91	0.14	-0.03	0.02	0.02
Grain numbers per ear (no.)	0.91	0.02	-0.06	0.07	0.05
Eigenvalue	3.17	1.97	1.87	1.63	1.30
Proportion/%	21.2	13.2	12.5	10.9	8.68
Cumulative proportion/%	21.2	34.3	46.8	57.7	66.5

In Shandan County, the first major component, described as ear characteristics, contributed at a rate of 36.2%. The second, third, fourth, and fifth primary component could be described as blade numbers, tiller numbers, flag leaf size, and stem length, which contributed at a rate of 13.599%, 12.272%, 8.148% and 6.879%, respectively. In Xinjin District, ear characteristics, flag leaf size, blade numbers, tiller numbers, and stem length contributed at a rate of 21.15%, 13.148%, 12.493%, 10.877% and 8.679%, respectively, based on the principal component analysis.

**Figure 5:**
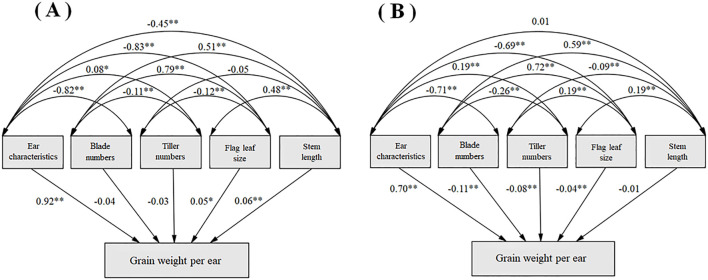
Structural equation modeling of the characteristics of indicators and grain weight per spike in Shandan County **(A)** and Xinjin District **(B)**.

A structural equation model is constructed between these five principal components and grain weight per ear (**Figure 5**). Ear characteristics had a strong direct contribution to grain weight in both planting regions. There is a significantly positive correlation between blade numbers and flag leaf size, while a significantly negative correlation between ear characteristics and both blade numbers and flag leaf size.

### 3.5 Multiple correspondence analysis of quantitative traits

**Figure 6:**
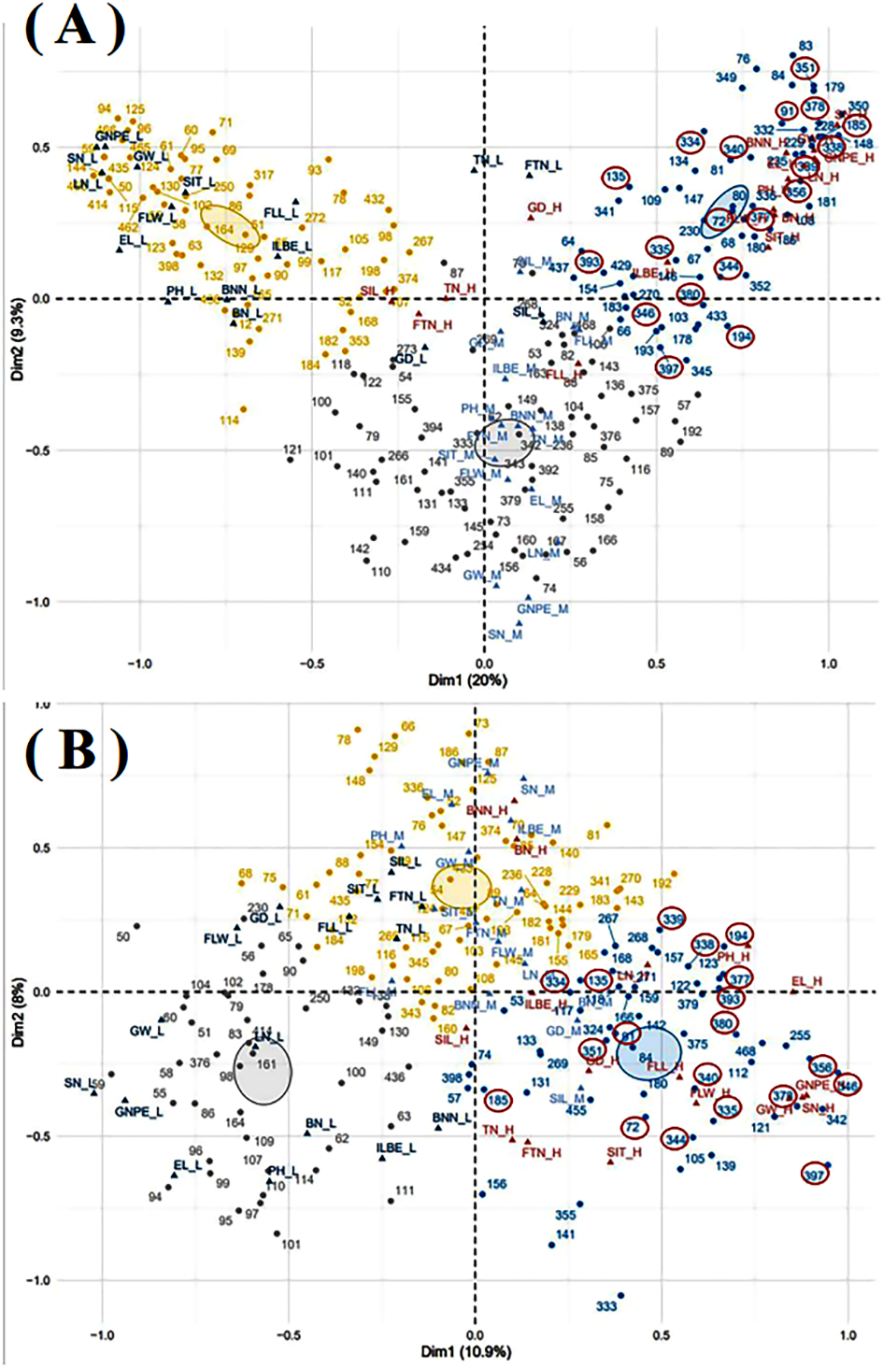
Multiple correspondence analysis in Shandan County **(A)** and Xinjin District **(B)**. GD, growth duration; BNN, branch node numbers; BN, blade numbers; SIT, the second internode thickness; SIL, the second internode length; ILBS, internode length below spike; PH, plant height; FLL, flag leaf length; FLW, flag leaf width; TN, tillers numbers; FTN, fertile tillers numbers; EL, ear length; LN, layer numbers; SN, spikelet numbers; GNPE, grain numbers per ear. The suffix letters after each quantitative trait, including H, M, and L, represent high, middle, and low, respectively. The resource numbers with a red circle were oat resources with a relatively high quantitative trait in both experimental sites.

The multiple correspondence analysis of sixteen quantitative traits was conducted for 183 germplasm resources in both experimental sites (**Figure 6**). Total nineteen oat germplasm resources, including 72, 91, 135, 185, 194, 334, 335, 338, 339, 340, 344, 346, 351, 356, 377, 378, 380, 393, and 397, showed the grain and biomass production potential in both experimental regions.

## 4 Discussion

Extensive germplasm resources constitute the fundamental basis for varietal breeding and improvement (Leišová-Svobodová et al., 2019; Tinker et al., 2009). Analyzing phenotypic trait diversity represents an efficient, intuitive, and practical approach to identify favorable genetic characteristics, thereby reducing breeding blindness and facilitating the rational utilization of germplasm to accelerate breeding progress (Subudhi et al., 2005). Quantitative traits demonstrate continuous variation patterns, being predominantly governed by multiple minor-effect polygenes and strongly influenced by environmental factors. Grain color showed the highest genetic diversity index in this study. Qualitative traits (including certain phenotypic characteristics) exhibit discrete segregation patterns, show greater genetic stability against environmental influences, and are typically controlled by major-effect genes (Kumar et al., 2017). In this study, the second branch node diameter, plant height, and flag leaf width exhibited maximal diversity, and this substantial variability provides a rich source of elite parental materials for varietal improvement (Feng et al., 2025; Dagnaw et al., 2023).

Correlation analysis of phenotypic traits serves as an effective method to streamline germplasm resource characterization and evaluation (Okasa et al., 2022). Our findings align with previous studies by Wu et al. (2023), who analyzed 180 accessions and similarly reported strong positive correlations between panicle length and several yield-related traits: layer numbers, spikelet numbers, grains per panicle, and grain weight. These consistent results confirm that longer panicles generally contain more layers and grains, consequently leading to increased grain yield (Wu et al., 2023). Furthermore, Chen et al. (2023b) documented a significant positive association between oat panicle length and plant height, which corroborates our current observations. Notably, we found that plant architecture traits (plant height and ear length) showed significant positive correlations with panicle characteristics (layer numbers, spikelet numbers, grains per ear, and grain weight). This consistent covariation pattern suggests these traits may share common genetic regulation, potentially being controlled by pleiotropic genes or closely linked loci (Tang et al., 2024).

Cluster analysis serves as a fundamental method for plant resource classification, varietal identification, and breeding research, primarily employed to investigate species genetic diversity (Zewodu et al., 2024; Chen et al., 2023a). This approach effectively elucidates distinct characteristics among different germplasm groups. 183 oat germplasm resources were classified into four major groups in this study. The clustering results revealed that identical accessions could appear in different clusters, reflecting how genetic variability arises from complex interactions between intrinsic genetic factors and extrinsic environmental conditions (Pittelkow et al., 2015). Notably, variations in edaphic and climatic factors significantly influence plant growth and development, causing germplasm resources to exhibit environmental plasticity in both genotypic and phenotypic traits (Nkhoma et al., 2020). These findings underscore the importance of conducting region-specific screening of superior oat germplasm, which holds substantial value for regional oat introduction and breeding programs.

PCA analysis elucidates inter-trait relationships while capturing comprehensive morphological characteristics of crops, thereby streamlining selection processes and enabling more scientific evaluation of oat germplasm resources (Abrar et al., 2024; Guo et al., 2024; Yano et al., 2019). We obtained five principal components, showing vegetative and reproductive growth performance, and the component structures showed remarkable consistency between regions, with minimal factor interference. At both locations, the first principal component predominantly represented grain yield-related traits, aligning with Mathias-Ramwell et al. (2023). This observation was further supported by Liang et al. (2021), who identified seven yield-associated traits (including grains per plant, grain weight, and ear characteristics) as major contributors to the first principal component in 590 hulled oat accessions. Path coefficient analysis reinforced these results, demonstrating strong direct effects of panicle characteristics on grain weight (**Figure 5**). These findings collectively emphasize that yield-related panicle traits should be prioritized in oat breeding programs.

## 5 Conclusion

This study evaluated the genetic diversity of 183 oat germplasm accessions, which were classified into four distinct groups through cluster analysis. Principal component analysis revealed that the sixteen quantitative traits could be reduced to five principal components, with ear characteristics demonstrating a strong direct influence on grain weight - identifying these as key selection criteria for future breeding programs. A total of nineteen oat resources were suggested to be the key resources with the potential for grain and biomass production by multiple correspondence analysis. However, since phenotypic traits are susceptible to environmental variations and subjective assessment, future research should integrate molecular marker technology with multi-environmental, multi-year evaluations. This integrated approach will provide more robust scientific foundations for oat genetic improvement, while simultaneously enhancing selection efficiency and accelerating breeding cycles.

## Data Availability

The original contributions presented in the study are included in the article/**Supplementary Material**. Further inquiries can be directed to the corresponding author/s.
